# Plasma substance P concentrations in patients undergoing general anesthesia: an objective marker associated with postoperative nausea and vomiting

**DOI:** 10.1186/s40981-016-0034-9

**Published:** 2016-06-02

**Authors:** Takako Kadota, Nami Kakuta, Yousuke T. Horikawa, Rie Tsutsumi, Takuro Oyama, Katsuya Tanaka, Yasuo M. Tsutsumi

**Affiliations:** 1 Department of Anesthesiology, Tokushima Univeristy, 3-18-15 Kuramoto, Tokushima, 770-8503 Japan; 2 Department of Pediatrics, Sharp Rees-Stealy Medical Group, San Diego, 92101 USA; 3 Department of Nutrition, Tokushima University, 3-18-15 Kuramoto, Tokushima, 770-8503 Japan

**Keywords:** PONV, Substance P, General anesthesia

## Abstract

**Background:**

This study investigated plasma concentrations of substance P (SP) in patients undergoing general anesthesia (GA) and postoperative nausea and vomiting (PONV). This prospective, observational, cohort study included 23 patients who underwent scheduled surgery under general anesthesia. Blood was collected from the radial artery at predetermined time points (15–30 mins prior anesthesia, 15–30 mins after surgery/GA, and 24 h after surgery). PONV, SP concentrations, risk factors, and analgesics used were measured.

**Findings:**

Nine of 23 patients experienced PONV. In patients without PONV, SP concentrations significantly decreased (*P* < 0.0001) at the end of surgery/GA, compared to baseline, and recovered at 24 h after surgery/GA (452.9 ± 146.2 vs. 666.9 ± 176.5 vs. 580.7 ± 168.6 pg/mL, respectively), whereas SP levels were unchanged during surgery/GA and increased at 24 hours after surgery (*P* = 0.020) in patients with PONV (726.1 ± 167.8 vs. 655.8 ± 168.0 vs. 779.7 ± 220.7 pg/mL, respectively).

**Conclusions:**

These finding suggest that SP levels may be utilized as an objective marker for PONV.

## Findings

### Introduction

Postoperative nausea and vomiting (PONV) is one of the most commonly reported adverse effects of anesthesia and surgery [1]. Various signaling mechanisms have been associated with PONV, including those related to opioid, dopamine, histamine, acetylcholine, serotonin, and substance P (SP) signaling [2, 3]. Some of these neurotransmitters have also been implicated as mediators of chemotherapy-induced nausea and vomiting (CINV) [4, 5]. These include, serotonin hydroxytryptamine type 3 (5-HT_3_) receptor antagonists and neurokinin-1 (NK1) receptor antagonist have been shown to be effective in the control of CINV [6].

SP is a neurotransmitter released from both the central nervous system and the peripheral nervous system afferent neurons, leading to nausea and vomiting by NK1 receptor activation [7]. Furthermore, SP is a known regulatory peptide that is excreted by enterochromafin cells and can act upon NK-1 receptors along the gastrointestinal tract inducing nausea and or vomiting [8]. A few studies have measured nausea- or vomiting-related plasma SP levels in patients receiving chemotherapy. A preliminary analysis by Higa et al., [4, 5] demonstrated increases in SP after administration of high doses of cisplatin and that increased SP was related to the emetic response. However, the relationship between PONV and concentrations of plasma SP in patients undergoing general anesthesia has not been determined. The aim of this study was to report plasma levels of SP in the perioperative period and its relationship to the incidence of PONV after general anesthesia (GA) during surgery.

## Methods

This pilot study, conducted from July through October 2014, was approved by the Human Research Ethics Committee of the Tokushima University registered in a clinical trials database (UMIN000015318). Written informed consent was obtained from all patients. We included 23 patients, ASA grade I-II, who were undergoing elective laparoscopic gynecologic surgical procedures with general anesthesia. Apfel scores were documented on all patients [9].

Anesthesia was induced with remifentanil (0.3 to 0.5 μg/kg/min), propofol (1.0 to 2.0 mg/kg), and rocuronium (0.8 mg/kg) and maintained with sevoflurane (1.0 to 2.0 %) in an oxygen and air mixture and remifentanil (0.1 to 0.5 μg/kg/min); incremental doses of rocuronium were used as necessary for muscle relaxation. Blood was collected from the radial artery at predetermined time points (15–30 min prior anesthesia, 15–30 min after surgery/GA prior to returning to the PACU, and 24 h after surgery). The plasma was immediately separated by centrifugation and stored at -20 °C until extraction. SP concentrations in samples of plasma were measured by ELISA, following the manufacturer protocol (Abcam, Cambridge, MA). All samples were assayed in duplicate.

PONV was determined by modified PONV scale developed by Wengritzky et al. [10] utilization of a nausea score between 0 and 3: 0 = absence of nausea, 1 = mild nausea, 2 = moderate nausea, 3 = severe nausea. A blinded physician who was not involved within the anesthesia visited and asked the patients 24 h after general anesthesia, the number of vomitus and the highest nausea score during the past 24 h. Any nausea score greater than one or any vomitus was considered PONV. Patients were given metoclopramide as a rescue antiemetic as needed.

### Statistical analysis

This pilot study without prior power calculation used a sample size of 23 similar to a study performed in CINV experiments [6]. Statistical analyses were performed using Prism version 6.0 software (GraphPad Software, Inc., La Jolla, CA). Data were expressed as the mean ± SD. Analysis was performed using the Mann–Whitney U test for independent data and the Friedman test for paired data. Values of *P* < 0.05 were considered statistically significant.

## Results

Twenty-three patients undergoing laparoscopic gynecological surgery with general anesthesia participated in this study, and all patients completed the study. Patient characteristics and individual results are presented in Table 1. Nine of 23 patients experienced PONV. Demographic data of two groups [PONV(-) and PONV(+) patients] are reported in Table 2.

**Table 1:** Patient characteristics and individual results

Patient number	Apfel score	ASA PS	Age (y)	BMI (kg/m^2^)	Baseline substance P (pg/mL)	PONV (yes/no)
1	3	I	42	22.0	530.7	No
2	2	II	49	19.0	492.5	No
3	3	II	64	22.2	782.8	No
4	2	II	51	23.3	808.1	No
5	2	I	38	21.5	870.1	Yes
6	2	I	41	27.1	423.7	No
7	2	II	56	26.4	620.4	No
8	3	I	41	24.0	726.3	Yes
9	2	II	51	21.4	862.5	No
10	3	I	34	19.1	868.3	No
11	2	II	55	25.5	731.2	Yes
12	2	II	29	18.3	870.1	No
13	2	I	26	21.2	620.1	No
14	2	I	33	21.7	907.6	No
15	3	II	32	28.7	525.9	Yes
16	3	II	42	24.7	480.1	Yes
17	4	II	37	19.5	837.9	Yes
18	1	II	50	24.9	470.8	No
19	3	I	40	20.1	617.9	Yes
20	3	II	68	23.7	737.5	Yes
21	3	I	45	29.3	1008.1	Yes
22	2	II	61	15.4	467.8	No
23	4	I	27	30.7	610.6	No

*ASA PS* American Society of Anesthesiologist Physical Status, *BMI* body mass index, *PONV* postoperative nausea and vomiting

**Table 2:** Patient demographics

	PONV(-) group	PONV(+) group
Patients	*n* = 14	*n* = 9
Duration of anesthesia, min	168 ± 29	159 ± 30
Intraoperative remifentanil, mg	1.9 ± 0.7	2.0 ± 0.4
Blood loss, mL	41 ± 10	50 ± 50
Fluid volume, mL	918 ± 183	866 ± 313
Risk factor		
Tobacco use (n)	3/14	1/9
History of motion sickness (n)	3/14	4/9
Postoperative opioids (n)	4/14	5/9
Woman (n)	14/14	9/9
Apfel score (0/1/2/3/4)	0/1/9/3/1	0/0/2/6/1
Analgesics used postoperatively		
Diclofenac	5 ± 13	9 ± 12
Flurbiprofen axetil	20 ± 42	18 ± 32
Pentazocine	3 ± 8	3 ± 18

Data are expressed as mean ± SD (range) or number of patients

*PONV* postoperative nausea and vomiting

**Fig. 1:**
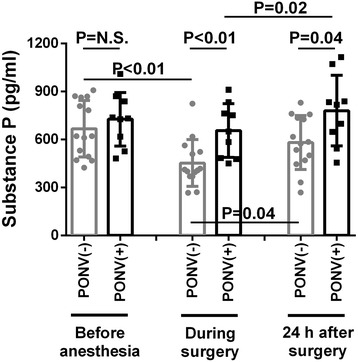
Plasma substance P concentrations in patients with and without postoperative nausea and vomiting (PONV). Calculated means at baseline before anesthesia, at the end of surgery, and 24 h after surgery were 607, 414, and 579 pg/mL, respectively for PONV(-) and 773, 631, and 879 pg/mL, respectively for PONV (+)

There were no differences in SP levels at baseline before anesthesia between PONV(-) and PONV(+) patients (666.9 ± 176.5 vs. 726.1 ± 167.8 pg/mL, respectively, *P* = 0.53), whereas SP levels of PONV(+) patients were higher than those of PONV(-) patients both at the end of surgery (655.8 ± 168.0 vs. 452.9 ± 146.2 pg/mL, respectively, *P* = 0.006) and 24 h after surgery (779.7 ± 220.7 vs. 580.7 ± 168.6 pg/mL, respectively, *P* = 0.039; Fig. 1). In the PONV(-) patient group, SP concentrations were significantly decreased at the end of surgery (*P* < 0.0001) when compared with pre-anesthesia levels, and returned to baseline levels 24 h after surgery (*P* = NS). However, SP concentrations in PONV(+) patients were not significantly decreased at the end of surgery and then continued to increase 24 h later when compared to post-operative levels (Fig. 1; *P* = 0.020). Four out of nine PONV (+) patients required rescue emetic following anesthesia.

## Discussion

Given the importance of SP in the pathogenesis of PONV, we hypothesized that SP could be used to identify patients with a high risk of developing PONV induced by general anesthesia. Analysis of data from our present study suggests that SP remains unchanged at the end of anesthesia and then increases over the subsequent 24 h correlating with PONV, whereas SP decreased at the end of anesthesia and recovered after 24 h in patients without PONV. These results suggest that peri and post-operative SP levels may help predict the need of prophylaxis and/or continued monitoring for PONV.

SP concentration as an indicator of pain intensity has been measured in other samples including cerebrospinal fluid (CSF), urine, and wound exudate [11–14]. To date, no studies have investigated plasma SP levels and its relationship to PONV after general anesthesia. However, SP signaling is not a novel mechanism in PONV. Numerous clinical studies have utilized antagonist against SP and have shown improvement over placebo [15] or even ondansetron [16]. Sjostrom et al., [11] demonstrated that CSF concentrations of SP following abdominal surgery and general anesthesia revealed no statistically significant changes following the procedure. However, SP concentrations have been shown to increase following spinal anesthesia in CSF [12] and in wound exudates [13]. Furthermore, urinary SP levels increased and then declined after epidural anesthesia [14]. Although these studies primarily did not investigate PONV, they investigated indirectly the effects of anesthesia on SP. Similarly, CINV investigations also revealed increases in SP following various chemotherapies [6].

Our results suggest that peri-operatively SP is not being expressed in PONV(-) patients as a significant decrease in SP is observed similar to non-cisplatin induced CINV [4], however, patients who expressed PONV continued to express SP peri- and post-operatively. Interestingly, all patients who developed PONV had Apfel scores of greater than or equal to 2, which correlates to a 39 % risk [9], although many with similar scores did not develop PONV. This novel, although preliminary, finding suggests that assessment of SP may result in a relatively accurate predictor of emetic symptoms induced by general anesthesia.

This study has some limitations. Higa et al., [6] measured serum SP levels in patients receiving chemotherapy, baseline SP levels were relatively higher among women and younger patients and are associated with higher risk of developing CINV. As our study had enrolled adult female patients undergoing laparoscopic gynecological surgery, differences may have been detected at peri- and post-operative periods. The results of this study might not be generalizable to other patient populations such as male, children, and other surgical procedures. Additional studies designed to investigate in the SP activity would be useful on this issue. Furthermore, our baseline SP values are higher than previously published values [6]. This is likely a result of different assaying systems and their sensitivities. Finally, nausea is a highly variable, patient-specific, and highly difficult symptom to quantify affecting our results. Utilizing a simple modified PONV scale allowed the incorporation of patients who may have not qualified as PONV in more stringent scales.

Specific receptors including opioid, dopamine, histamine, acetylcholine, serotonin, and SP are known to be associated PONV [2, 3]. In the present study, however, we only focused on the activity of SP levels. Another possible limitation is that after binding to the NK1 receptors, SP regulates many biological functions. These mediators may have affected the activity of SP levels. A larger sample size is required in order to determine the clinical profile of SP.

## Conclusions

Predicting whether a patient will have any adverse reactions to general anesthesia is extremely difficult and limited. Our results suggest that SP levels may increase the ability to correctly predict patients with PONV. This preliminary study suggests a role in continued measuring of SP levels post-operatively in order to objectively monitor PONV.
